# Supplementary figures and images for: Cognitive cascades: How to model (and potentially counter) the spread of fake news
Source: PLoS One. 2022 Jan 7;17(1):e0261811. doi: 10.1371/journal.pone.0261811 (PMC8740964; doi:10.1371/journal.pone.0261811)

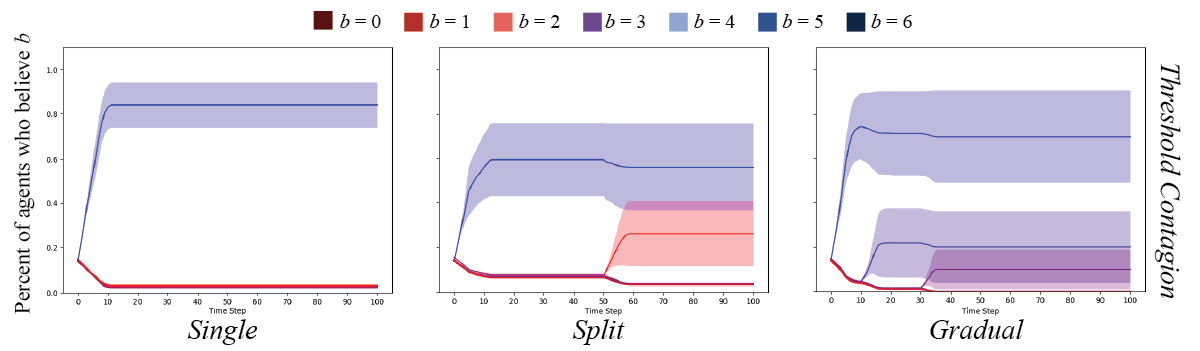

Supplement: S1 Fig — Proportional threshold contagion results over ten iterations of a Watts-Strogatz small world network with N = 500, initial neighbors k = 5, and rewiring chance ρ = 0.5. Graphs show the mean percent of agents who believe b ∈ B, color coded by b value, plotted against time step. Shaded portions show variance over iterations. (PNG) [file pone.0261811.s007.png]

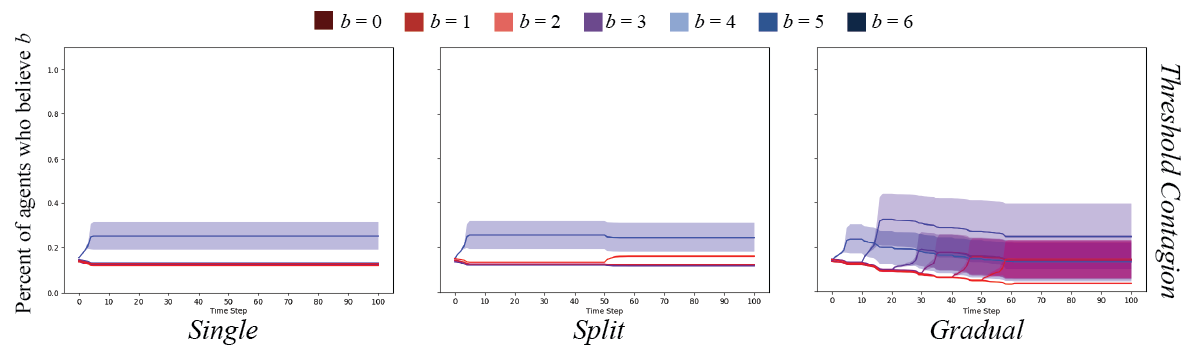

Supplement: S2 Fig — Proportional threshold contagion results over ten iterations of a Barabási-Albert preferential attachment network with N = 500, and added edges m = 3. Graphs show the mean percent of agents who believe b ∈ B, color coded by b value, plotted against time step. Shaded portions show variance over iterations. (PNG) [file pone.0261811.s008.png]

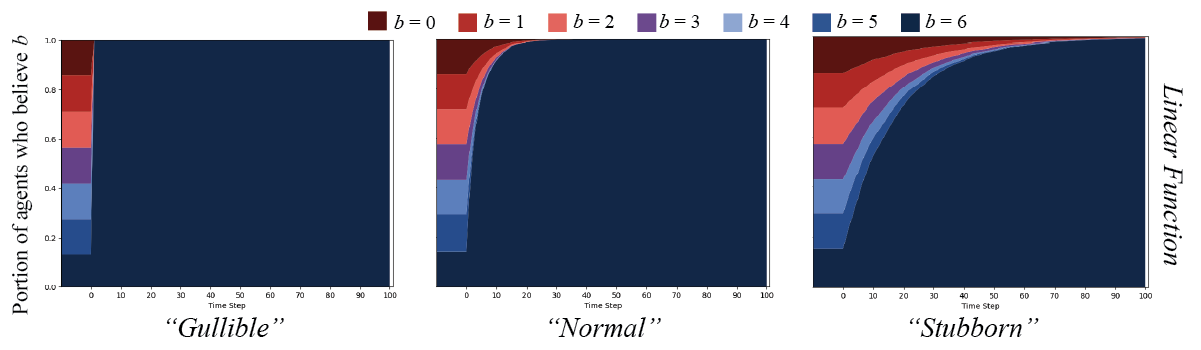

Supplement: S3 Fig — The single message set on an ER random graph, N = 250, ρ = 0.05, for agents updating their beliefs based on the inverse linear cognitive contagion function in Eq (5) in the main paper. Graphs display percent of agents who believe B with strength b over time. The left graph shows agents parameterized to be “gullible” (γ = 1, α = 0); the middle shows “normal” agents (γ = 1, α = 1), and the right, “stubborn” agents (γ = 10, α = 20). (PNG) [file pone.0261811.s009.png]

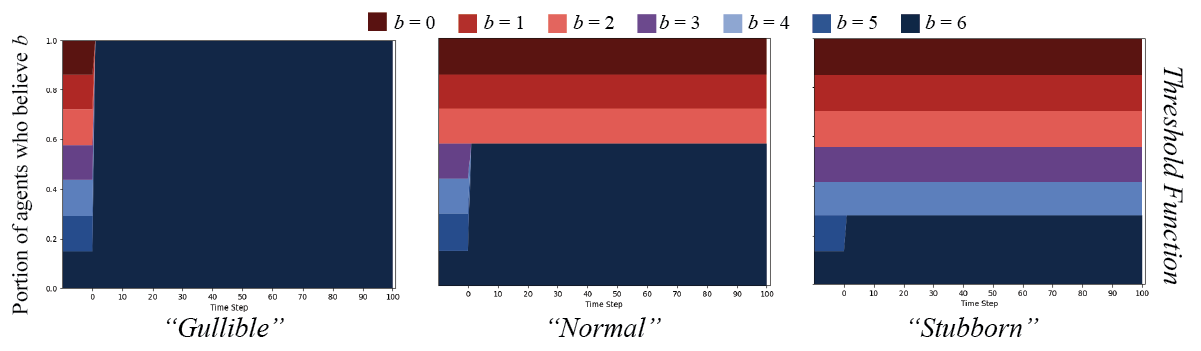

Supplement: S4 Fig — The single message set on an ER random graph, N = 250, ρ = 0.05, for agents updating their beliefs based on the threshold cognitive contagion function in Eq (2) in the main paper. Graphs display percent of agents who believe B with strength b over time. The left graph shows agents parameterized to be “gullible” (γ = 6); the middle shows “normal” agents (γ = 3); and the right, “stubborn” agents (γ = 1). (PNG) [file pone.0261811.s010.png]

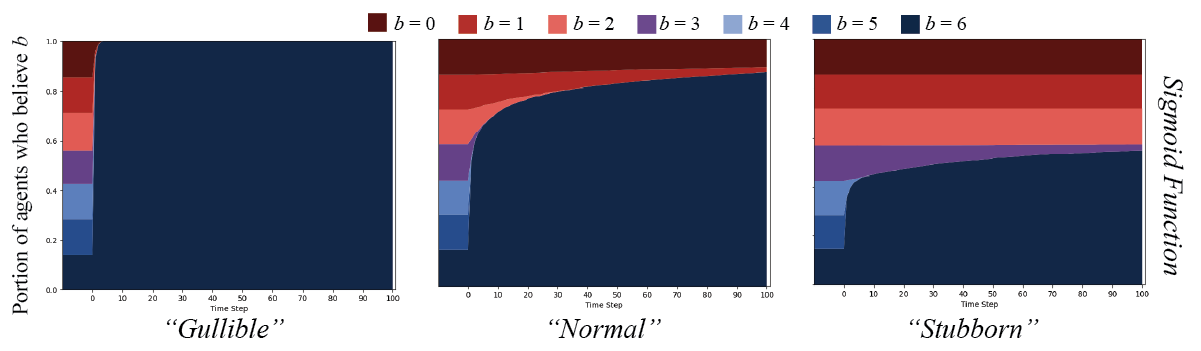

Supplement: S5 Fig — The single message set on an ER random graph, N = 250, ρ = 0.05, for agents updating their beliefs based on the sigmoid cognitive contagion function in Eq (6) in the main paper. Graphs display percent of agents who believe B with strength b over time. The left graph shows agents parameterized to be “gullible” (α = 1, γ = 7), the middle shows “normal” agents (α = 2, γ = 3), and the right, “stubborn” agents (α = 4, γ = 2). (PNG) [file pone.0261811.s011.png]

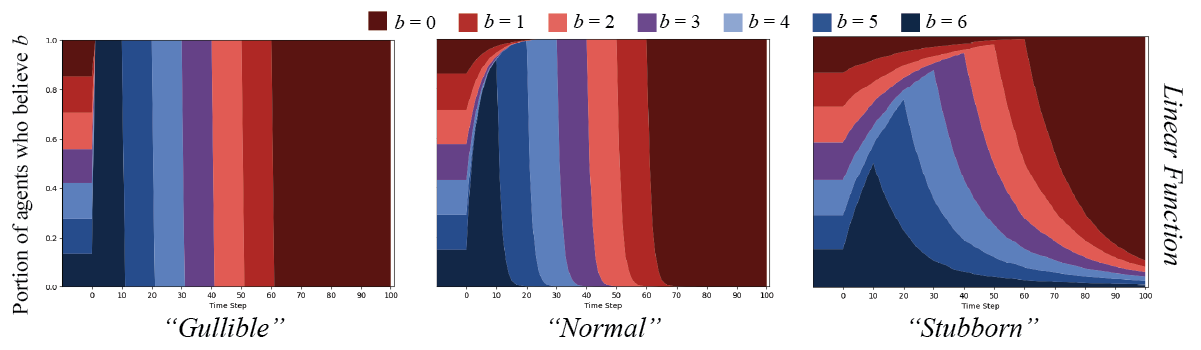

Supplement: S6 Fig — The gradual message set on an ER random graph, N = 250, ρ = 0.05, for agents updating their beliefs based on the inverse linear cognitive contagion function in Eq (5) in the main paper. Graphs display percent of agents who believe B with strength b over time. The left graph shows agents parameterized to be “gullible” (γ = 1, α = 0); the middle shows “normal” agents (γ = 1, α = 1), and the right, “stubborn” agents (γ = 10, α = 20). (PNG) [file pone.0261811.s012.png]

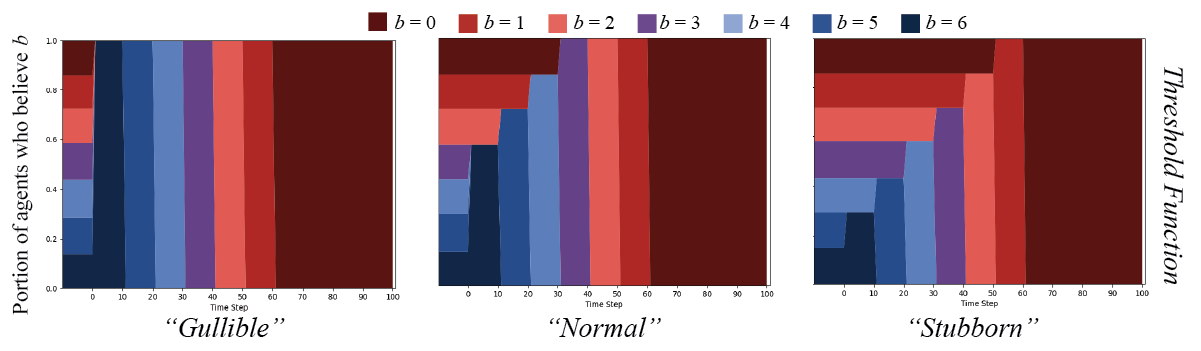

Supplement: S7 Fig — The gradual message set on an ER random graph, N = 250, ρ = 0.05, for agents updating their beliefs based on the threshold cognitive contagion function in Eq (2) in the main paper. Graphs display percent of agents who believe B with strength b over time. The left graph shows agents parameterized to be “gullible” (γ = 6); the middle shows “normal” agents (γ = 3); and the right, “stubborn” agents (γ = 1). (PNG) [file pone.0261811.s013.png]

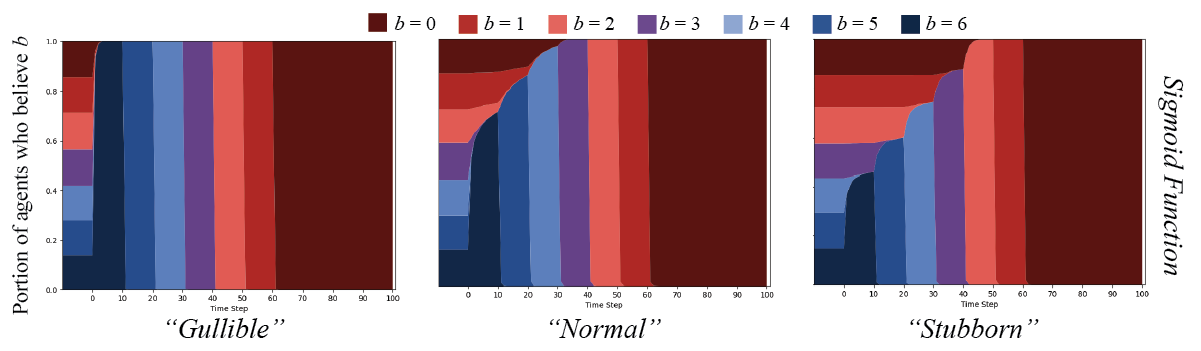

Supplement: S8 Fig — The gradual message set on an ER random graph, N = 250, ρ = 0.05, for agents updating their beliefs based on the sigmoid cognitive contagion function in Eq (6) in the main paper. Graphs display percent of agents who believe B with strength b over time. The left graph shows agents parameterized to be “gullible” (α = 1, γ = 7), the middle shows “normal” agents (α = 2, γ = 3), and the right, “stubborn” agents (α = 4, γ = 2). (PNG) [file pone.0261811.s014.png]

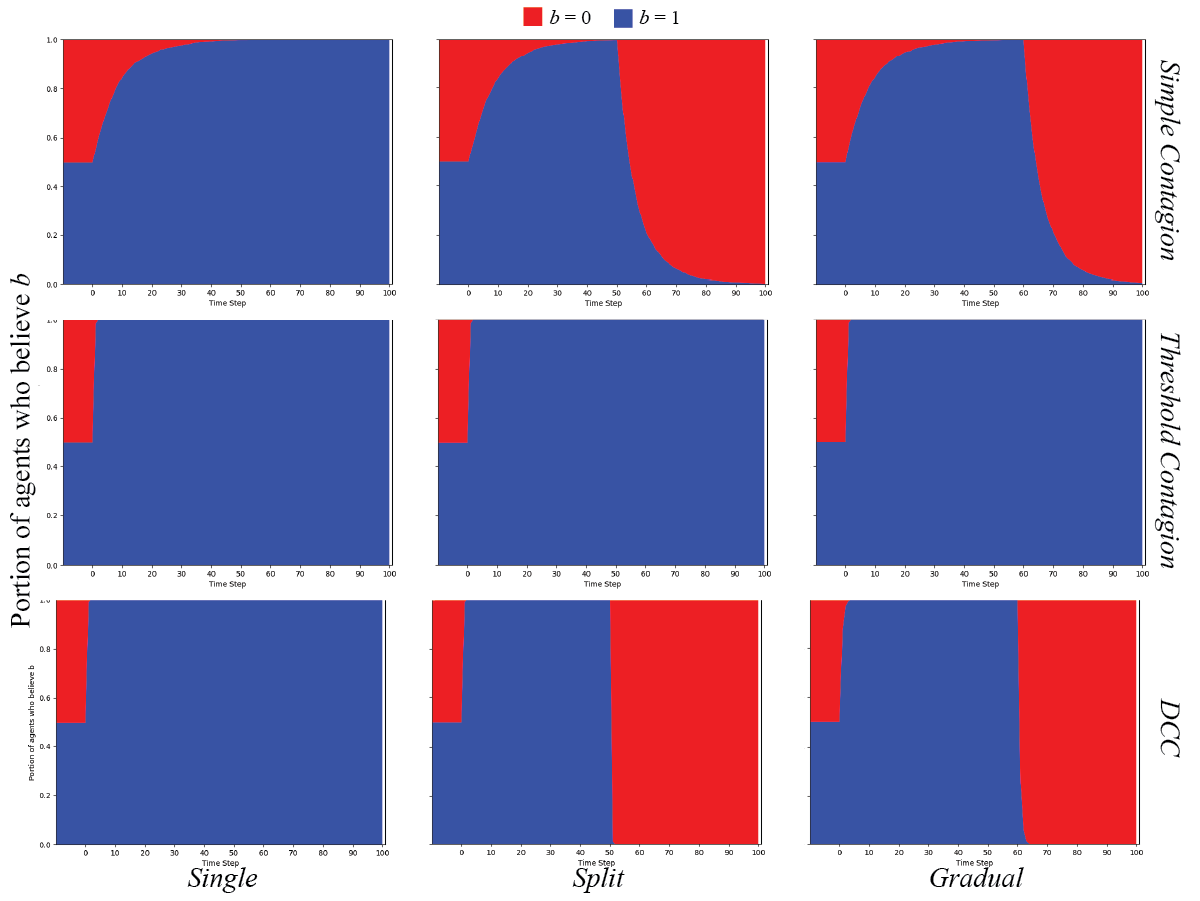

Supplement: S9 Fig — The single, split, and gradual message sets on a Barabási-Albert preferential attachment graph, N = 500, and added edges m = 3. Graphs display percent of agents who believe some b in B={b,0≤b≤2},b∈Z. (PNG) [file pone.0261811.s015.png]

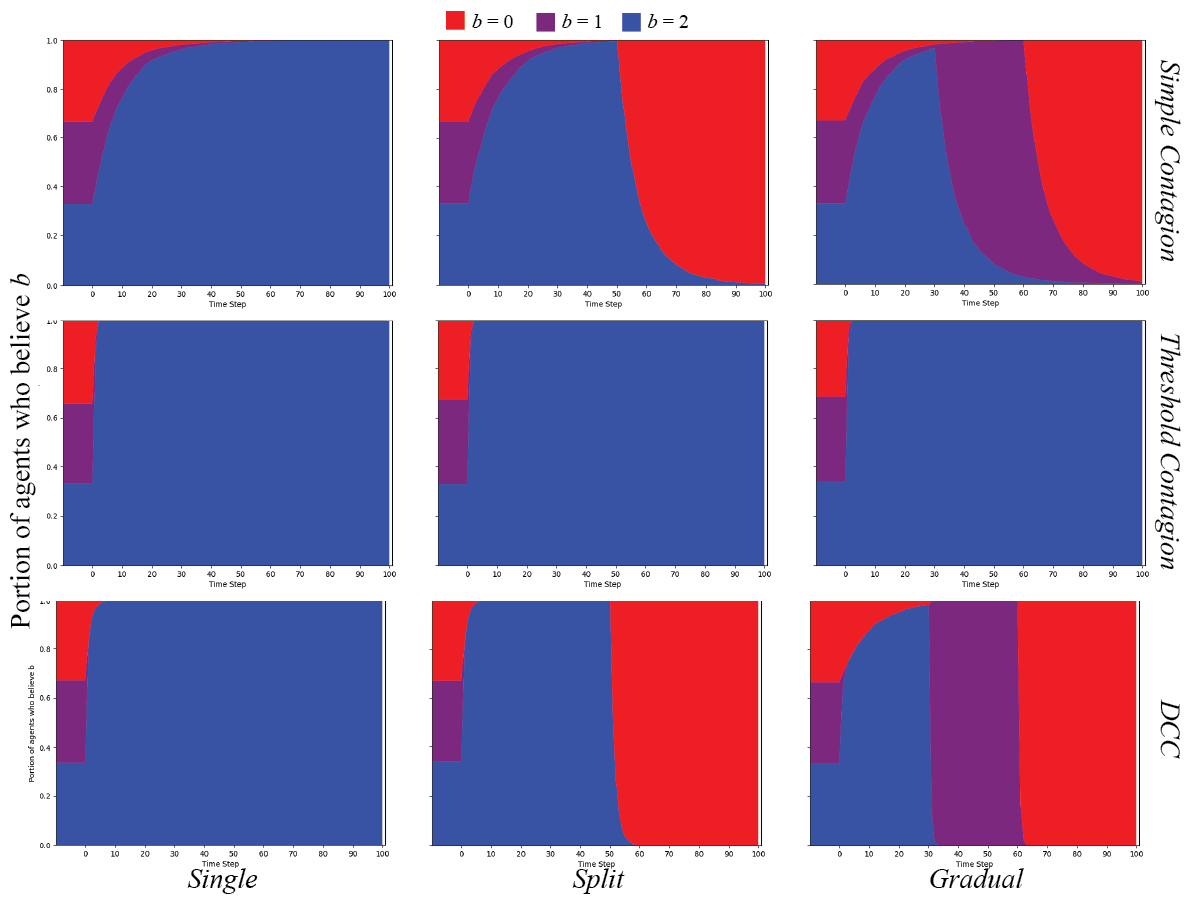

Supplement: S10 Fig — The single, split, and gradual message sets on a Barabási-Albert preferential attachment graph, N = 500, and added edges m = 3. Graphs display percent of agents who believe some b in B={b,0≤b≤3},b∈Z. (PNG) [file pone.0261811.s016.png]

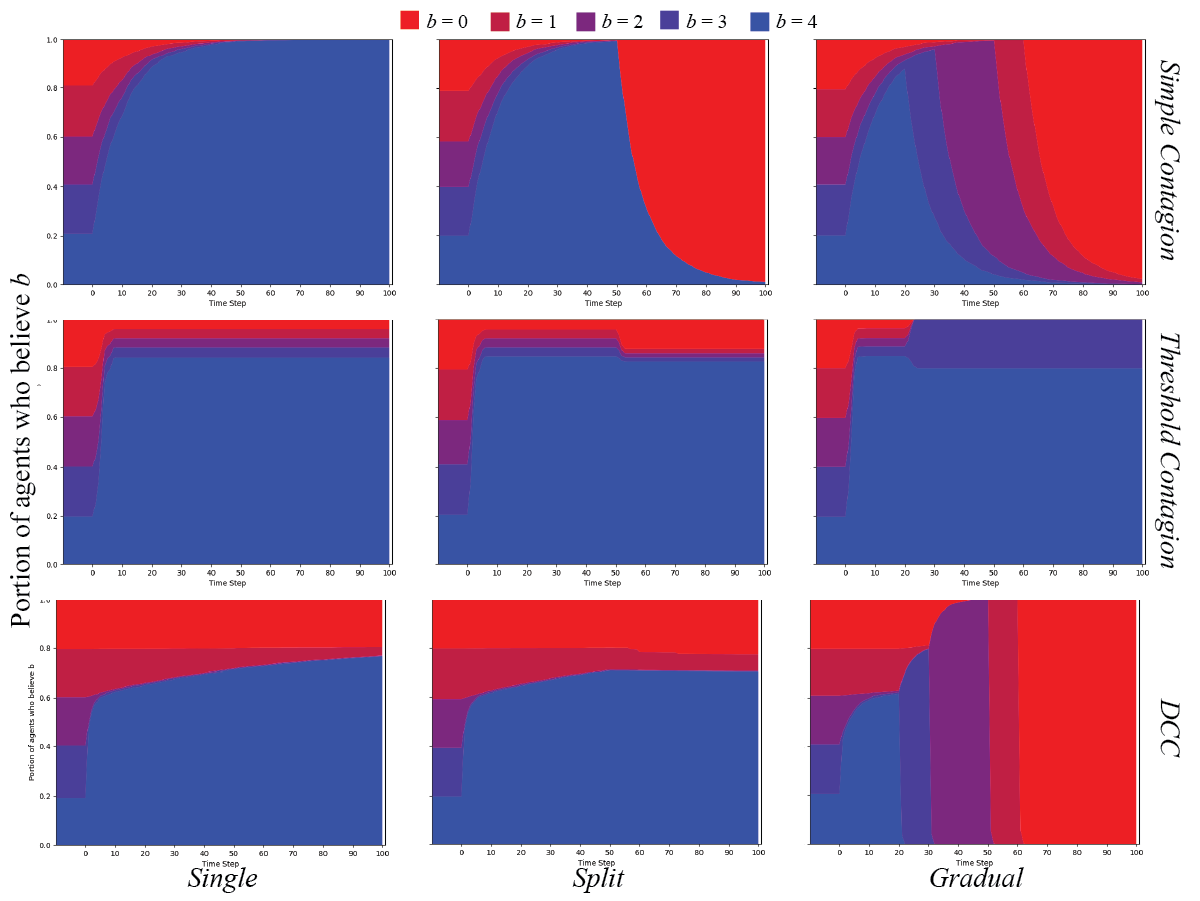

Supplement: S11 Fig — The single, split, and gradual message sets on a Barabási-Albert preferential attachment graph, N = 500, and added edges m = 3. Graphs display percent of agents who believe some b in B={b,0≤b≤5},b∈Z. (PNG) [file pone.0261811.s017.png]

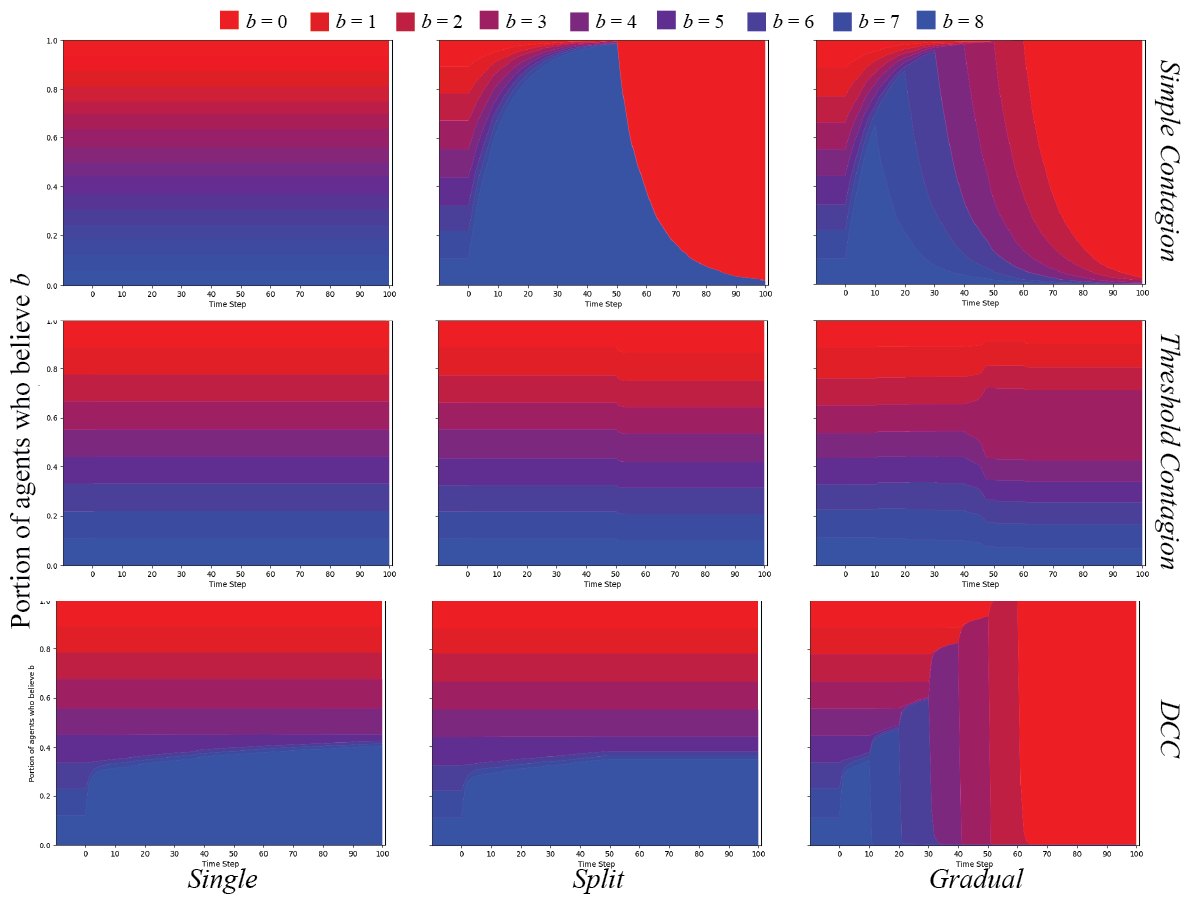

Supplement: S12 Fig — The single, split, and gradual message sets on a Barabási-Albert preferential attachment graph, N = 500, and added edges m = 3. Graphs display percent of agents who believe some b in B={b,0≤b≤9},b∈Z. (PNG) [file pone.0261811.s018.png]

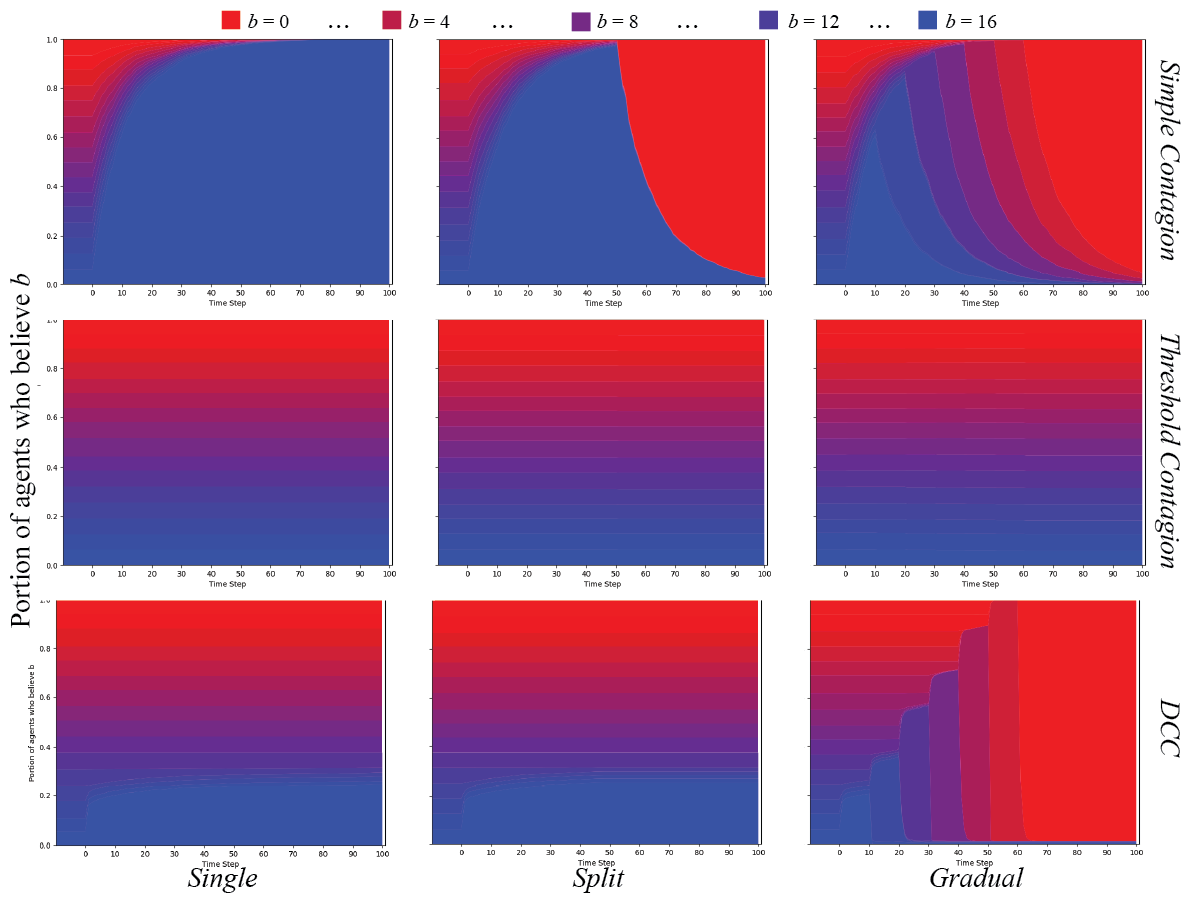

Supplement: S13 Fig — The single, split, and gradual message sets on a Barabási-Albert preferential attachment graph, N = 500, and added edges m = 3. Graphs display percent of agents who believe some b in B={b,0≤b≤16},b∈Z. (PNG) [file pone.0261811.s019.png]

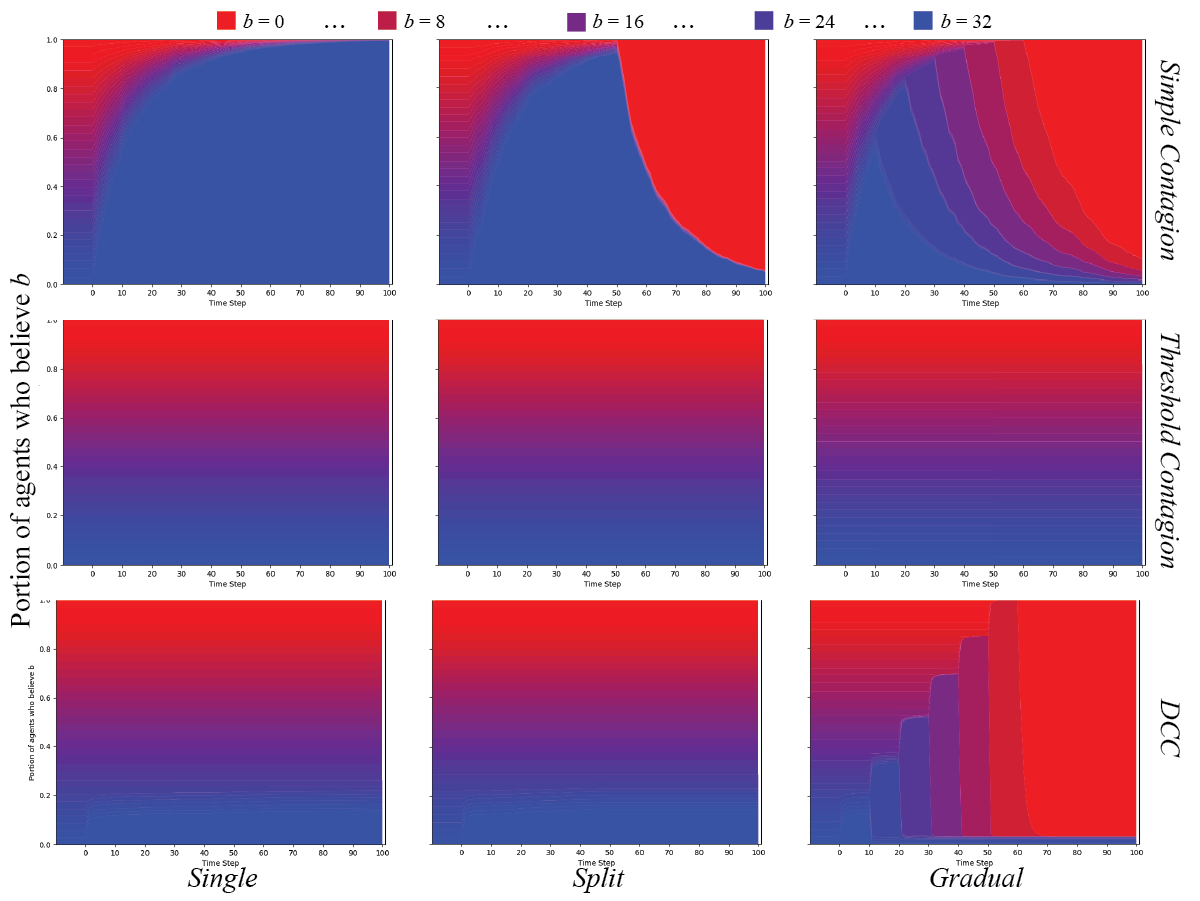

Supplement: S14 Fig — The single, split, and gradual message sets on a Barabási-Albert preferential attachment graph, N = 500, and added edges m = 3. Graphs display percent of agents who believe some b in B={b,0≤b≤32},b∈Z. (PNG) [file pone.0261811.s020.png]

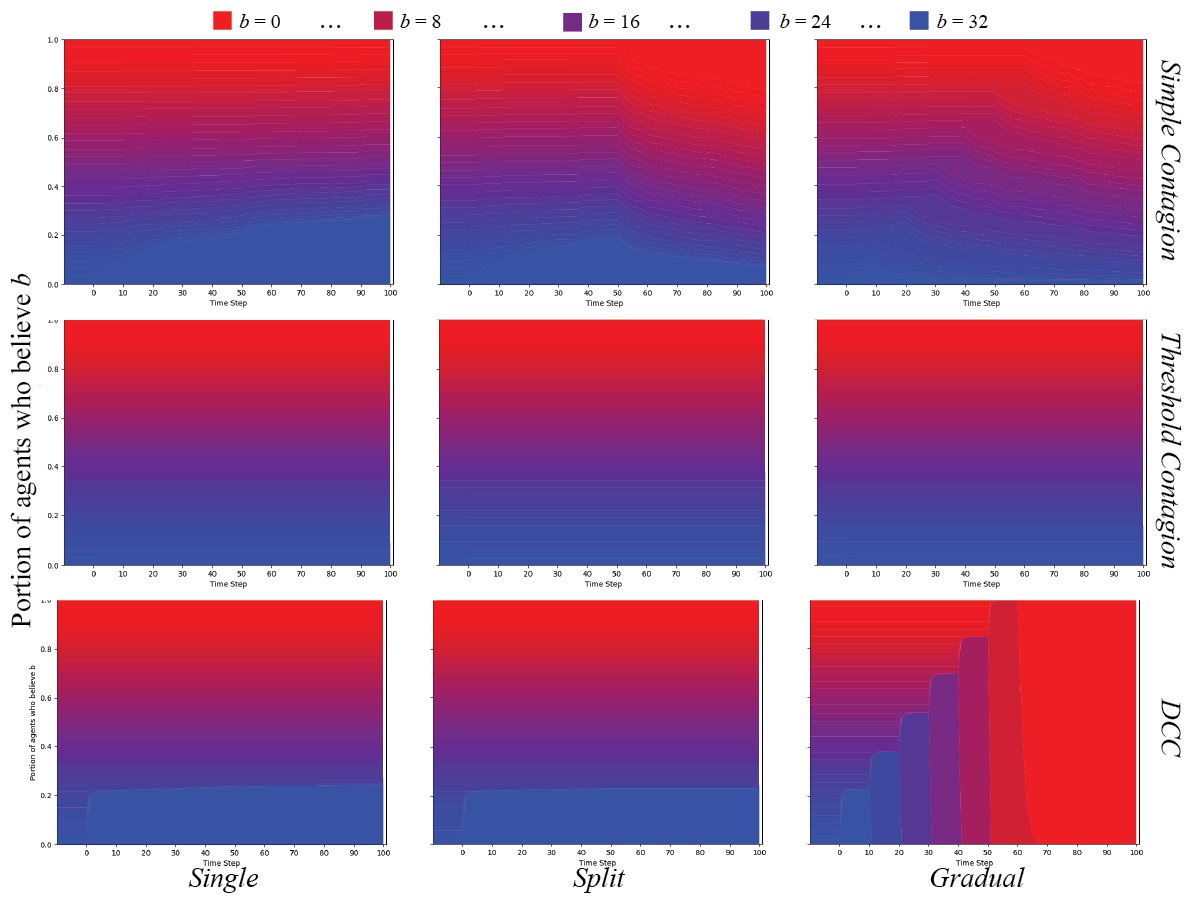

Supplement: S15 Fig — The single, split, and gradual message sets on a Multiplicative Attribute Graph, N = 500, and Θ generated from the same formula in Eq (8) in the main paper—i.e. one that brings about high levels of homophily so agents would rarely connect to agents more than 3 belief values away from them. Graphs display percent of agents who believe some b in B={b,0≤b≤32},b∈Z. (PNG) [file pone.0261811.s021.png]

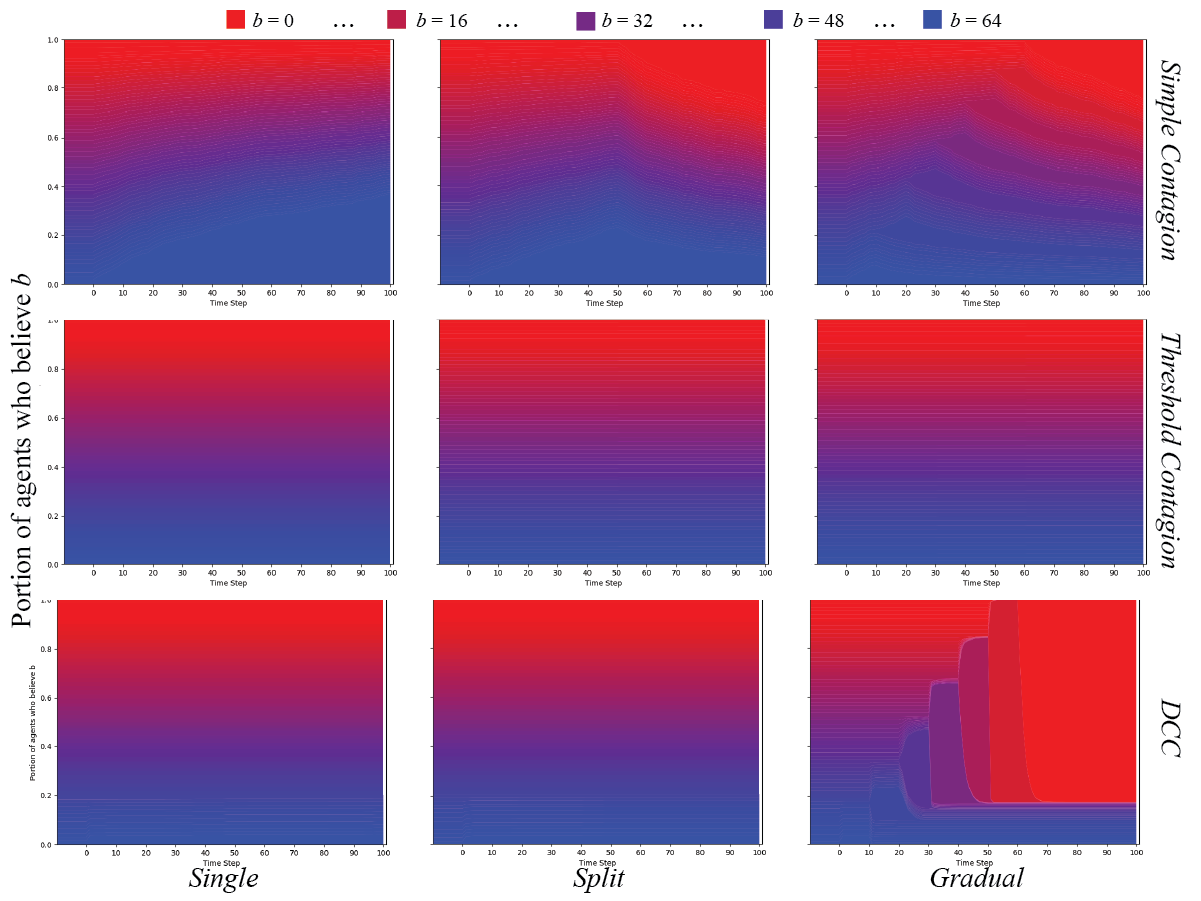

Supplement: S16 Fig — The single, split, and gradual message sets on a Watts-Strogatz small world network with N = 500, initial neighbors k = 5, and rewiring chance ρ = 0.5. Graphs display percent of agents who believe some b in B={b,0≤b≤64},b∈Z. (PNG) [file pone.0261811.s022.png]

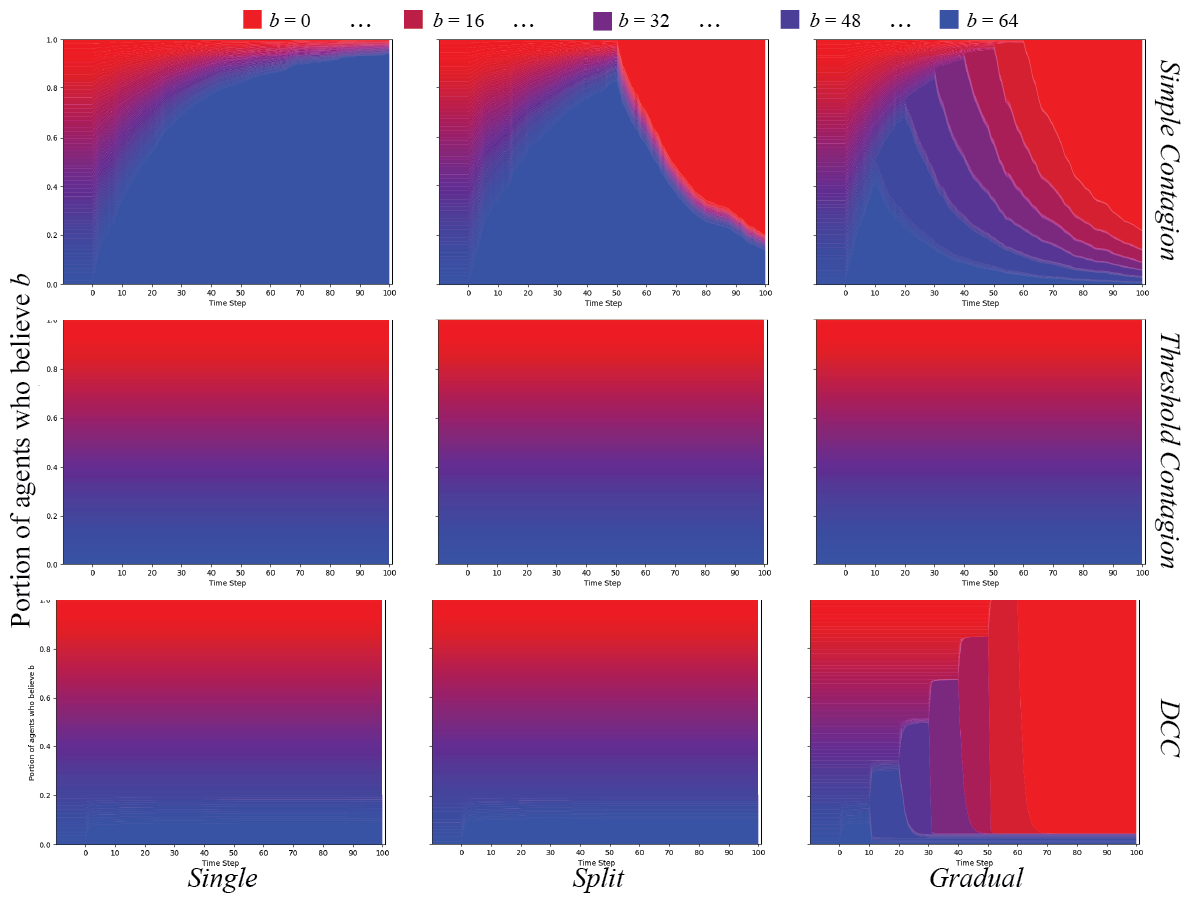

Supplement: S17 Fig — The single, split, and gradual message sets on a Barabási-Albert preferential attachment graph, N = 500, and added edges m = 3. Graphs display percent of agents who believe some b in B={b,0≤b≤64},b∈Z. (PNG) [file pone.0261811.s023.png]

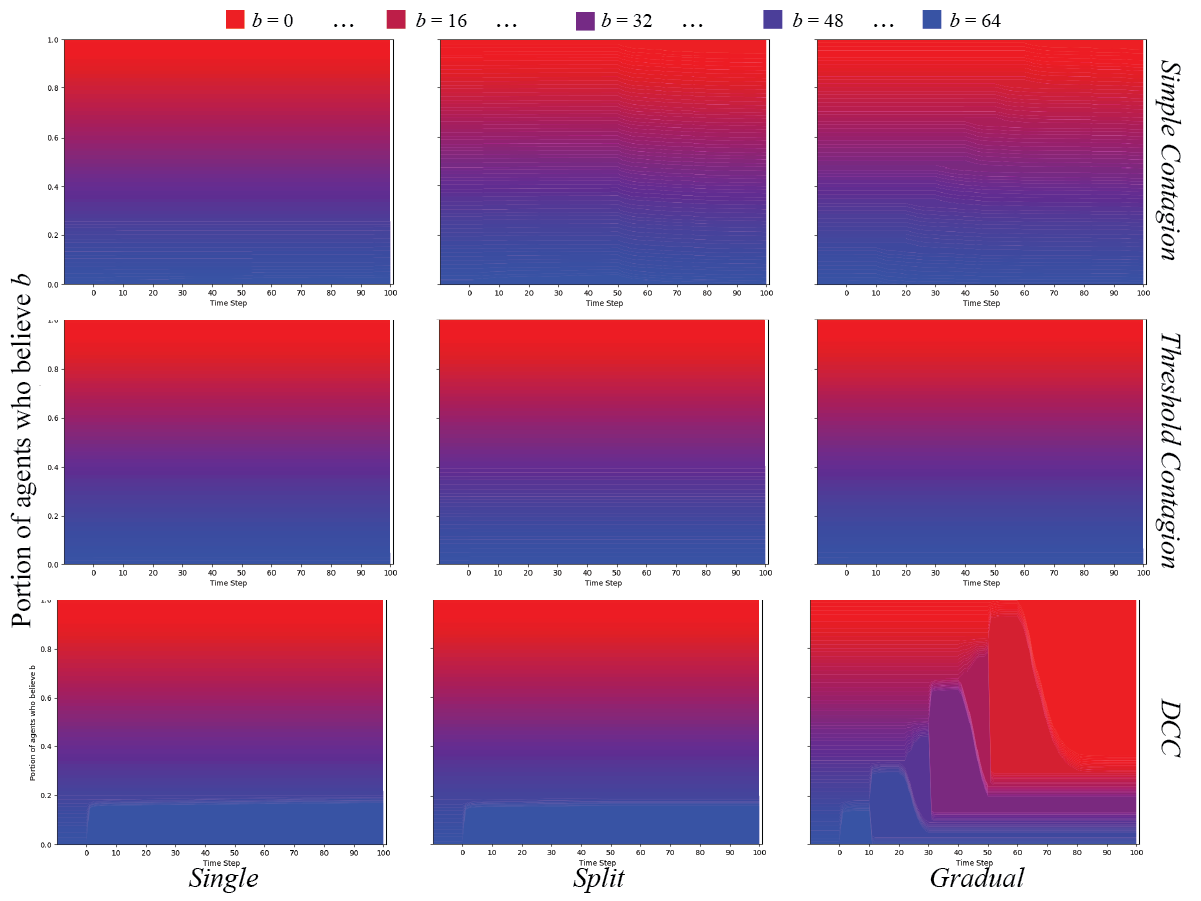

Supplement: S18 Fig — The single, split, and gradual message sets on a Multiplicative Attribute Graph, N = 500, and Θ generated from the same formula in Eq (8) in the main paper—i.e. one that brings about high levels of homophily so agents would rarely connect to agents more than 3 belief values away from them. Graphs display percent of agents who believe some b in B={b,0≤b≤64},b∈Z. (PNG) [file pone.0261811.s024.png]

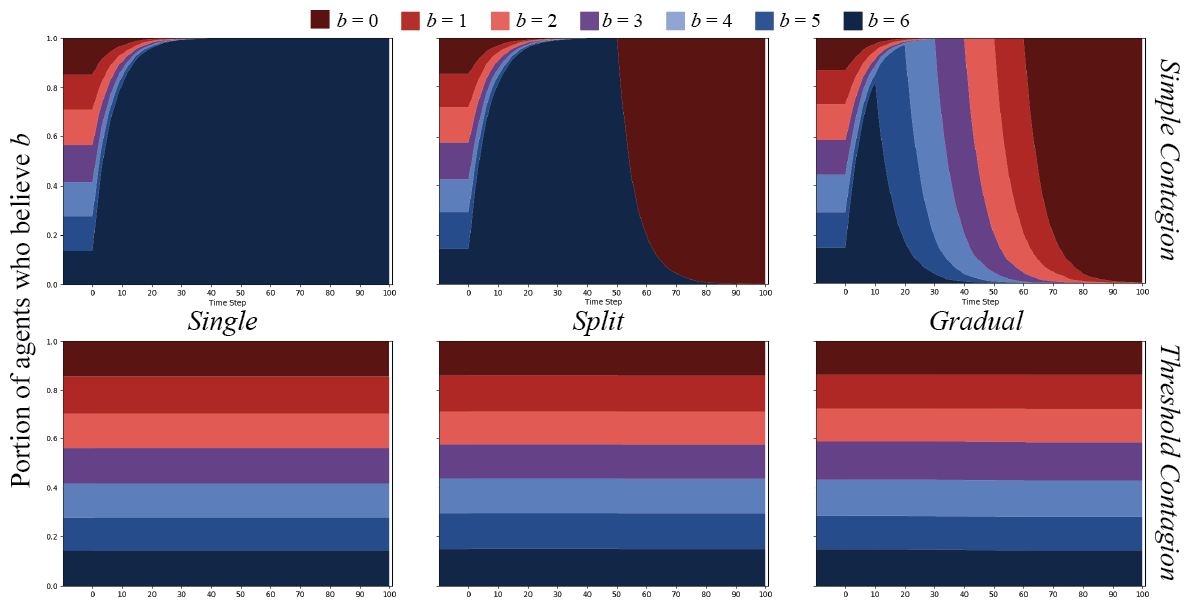

Supplement: S19 Fig — Simple (top row) and proportional threshold (bottom row) contagion on ER random networks with N = 500, and connection chance ρ = 0.05. Graphs show the percent of agents who believe B with strength b over time. (PNG) [file pone.0261811.s025.png]

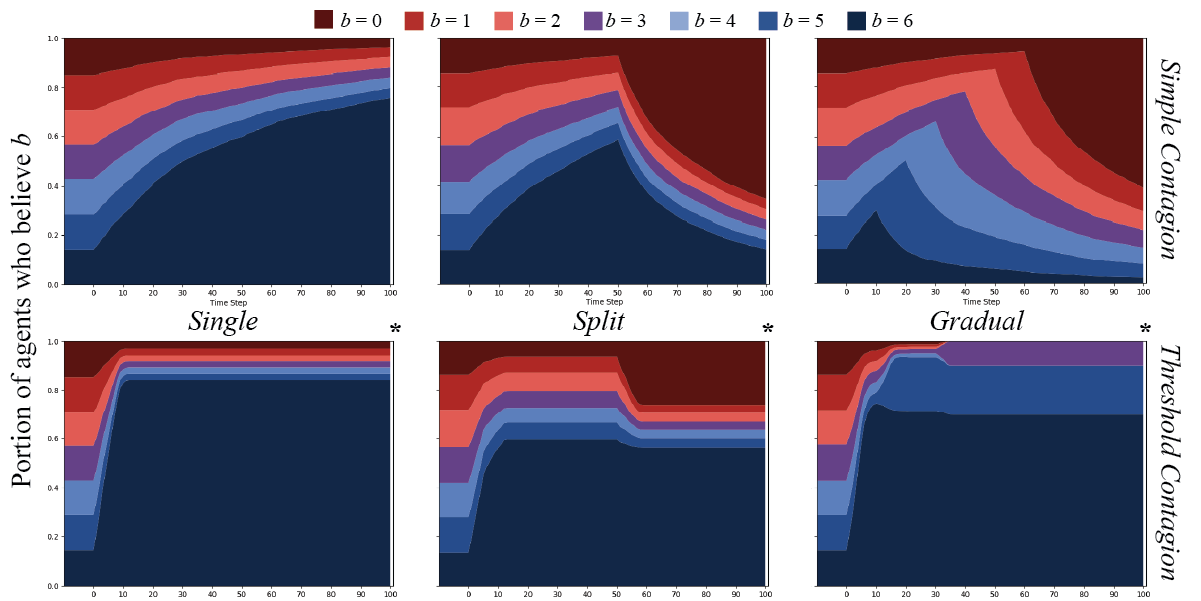

Supplement: S20 Fig — Simple (top row) and complex (bottom row) contagion on a Watts-Strogatz small world network with N = 500, initial neighbors k = 5, and rewiring chance ρ = 0.5. Graphs show the percent of agents who believe B with strength b over time. Asterisks (*) denote these contagions had significant variance over simulation iterations. (PNG) [file pone.0261811.s026.png]

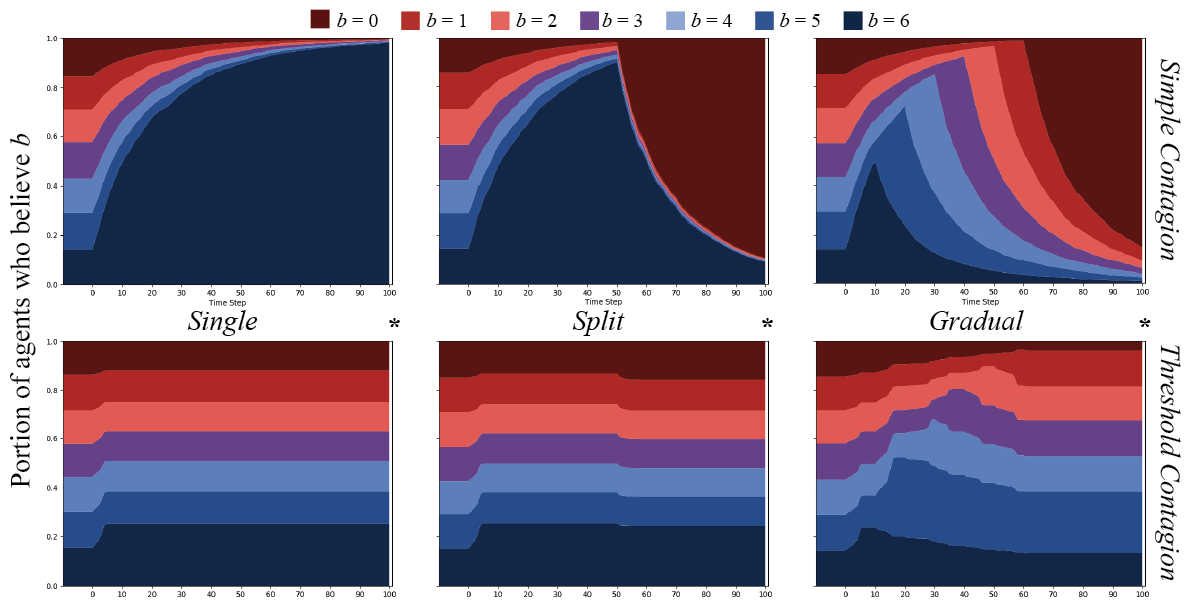

Supplement: S21 Fig — Simple (top row) and proportional threshold (bottom row) contagion on Barabási-Albert preferential attachment networks with N = 500, and added edges m = 3. Graphs show the percent of agents who believe B with strength b over time. Asterisks (*) denote these contagions had significant variance over simulation iterations. (PNG) [file pone.0261811.s027.png]

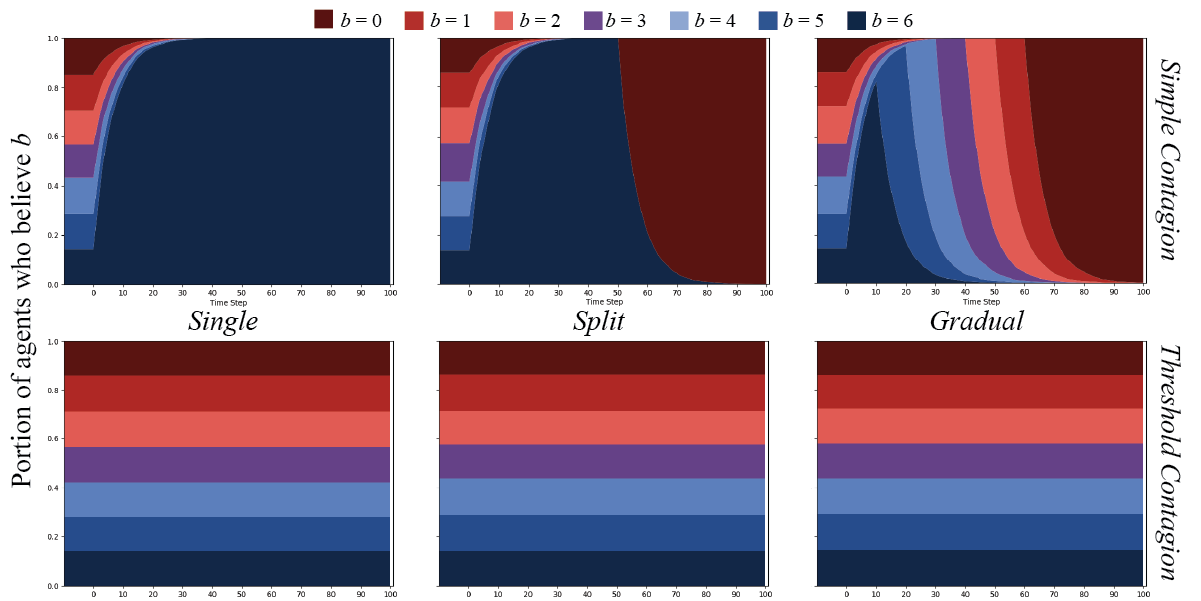

Supplement: S22 Fig — Simple (top row) and proportional threshold (bottom row) contagion on a homophilic MAG networks with N = 500, and Θb detailed in Eq (8). Graphs show the percent of agents who believe B with strength b over time. (PNG) [file pone.0261811.s028.png]

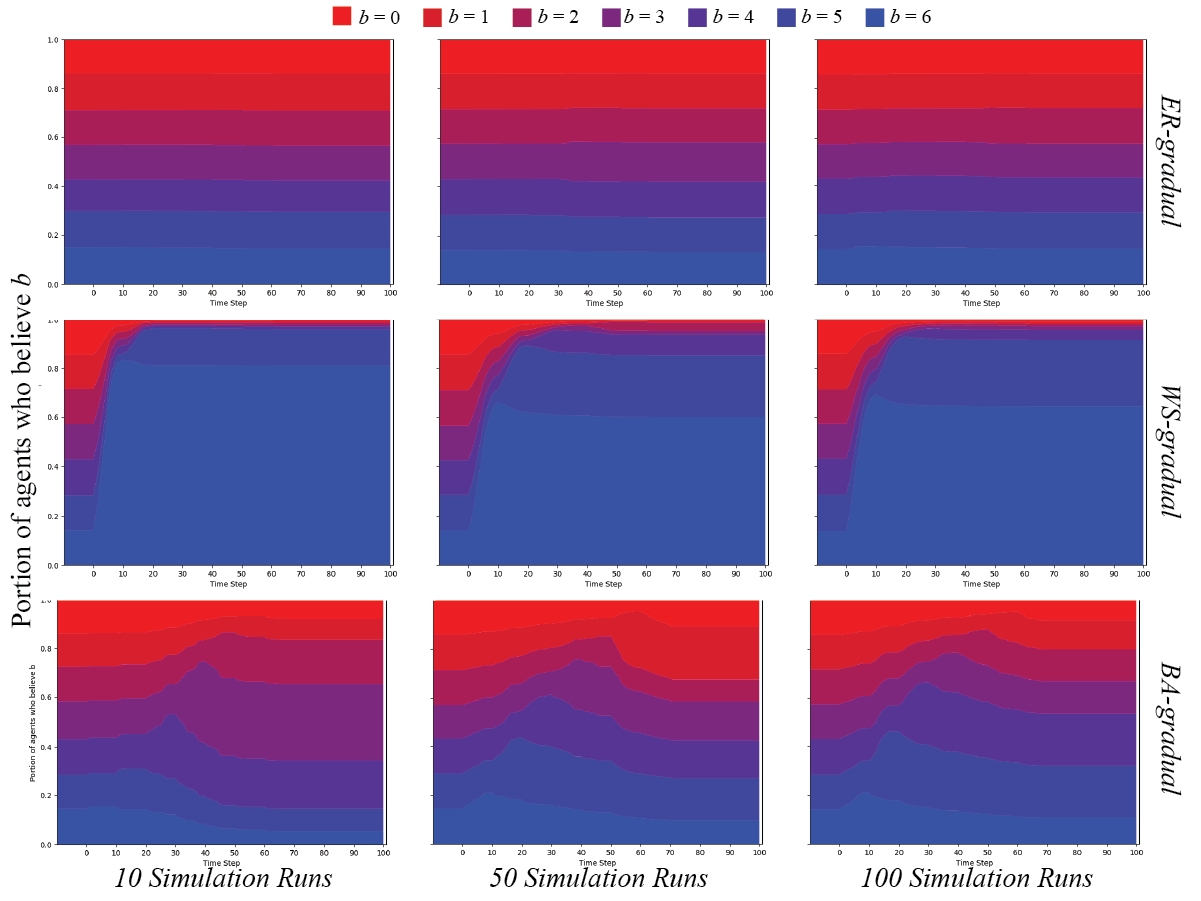

Supplement: S23 Fig — Graphical results of contagion cascades across 10, 50, and 100 simulation runs for specific graph-message set combinations. Results displayed are those which yielded the lowest correlation scores between simulation run counts. (PNG) [file pone.0261811.s029.png]
