# Supplementary material for: Cognitive cascades: How to model (and potentially counter) the spread of fake news
Source: PLoS One. 2022 Jan 7;17(1):e0261811. doi: 10.1371/journal.pone.0261811 (PMC8740964; doi:10.1371/journal.pone.0261811)
Supplement: Attachment — Submitted filename: Review of PONE-D-21-06938.pdf [file pone.0261811.s030.pdf]

Review of PONE-D-21-06938: Cognitive contagion: How to model (and potentially counter) the spread of fake news

This paper presents a model of diffusion that is grounded in the cognition of each agents. By comparing different graph types to the exposure to information/beliefs coming from an institutional source, the authors try to show how adding complexity to the definition of an agent may lead to map more closely what happened, for example, with beliefs around COVID-19.

While certainly interesting and timely, the article presents a series of concerns that make the message a bit less effective than it could be. The most relevant concerns I had are those around (a) the claim that more complex agents is something "new" of this model, while the ABM community has been discussing it since its beginnings, (b) the use of ABM (especially the "why" ABM), (c) the complete lack of reference to description and reporting standards in presenting the ABM, and (d) the report of results shows that you are probably trying to do too much with just one paper.

I have detailed what I mean by this in the following points, I hope you find them useful. I enjoyed reading your paper. Best of luck with your research!

1. You may want to make sure that the abstract does not lean too much on text that is already in the paper. Sometimes it makes sense to express the same concepts differently.

2. Your introduction reads well and I agree with you on the excessive price that diffusion models pay to epidemiology, especially the spread of diseases. In fact, most models of diffusion even use the same categories — e.g., immune, susceptible, infected — even though this may not have much sense in a social environment. I believe the broader category for these models is that of "threshold models of diffusion" (from Granovetter 1978 til Rosenkopf & Abrahamson 1999) and it would be a good idea for you to also acknowledge this literature more than you already do.

   Granovetter M (1978) Threshold models of collective behavior. Am J Sociol 83(6):1420–1443.

   Rosenkopf L, Abrahamson E (1999) Modeling reputational and informational influences in threshold models of bandwagon innovation diffusion. Comput Math Organiz Theory 5(4):361–384.

3. I have been working on diffusion model for quite some time now and I have found the literature review by Kiesling et al (2012) — a work that appears in your reference list — very much informative and well researched. They present and categorize diffusion models by considering what agent-based modeling brings to the theory. Even though they do not explicitly mention cognition, they do refer to individual characteristics (e.g., psychological, social) that affect the diffusion process. Moreover, there have been agent-based models (ABMs) of diffusion with a cognitive backbone (Secchi & Gullekson 2016). I am writing this because I do not think you can claim that you are introducing "a new class of models" (p.2, line 25). In fact, this class of agent-based models that focus on individual attitudes, dispositions, perceptions as a way to study diffusion are very much the reason why scientists have turned to agent-based models. Besides this point, it is my understanding that

PLOS is not interested in novelty as such, scientific robustness would be enough to grant publication. In short, I do agree with your approach and I think it is sound but, at the same time, I do not see the need to oversell. This is both because what you claim as unique is part of the ABM approach and because PLOS journals are not the place where these claims make a difference.

Kiesling E, Günther M, Stummer C, Wakolbinger LM (2012) Agent-based simulation of innovation diffusion: a review. CEJOR 20(2):183–230.

Secchi D, Gullekson NL (2016) Individual and organizational conditions for the emergence and evolution of bandwagons. Comput Math Organiz Theory 22(1):88–133.

4. Your reference for agent-based social systems [37] is not what I was expecting. I was almost certain to find one of the publications from Macal and North… I may be mistaken here.

5. Your call for simplicity in ABM (p.3, lines 67-75) is a bit puzzling, perhaps since you cite a cellular automata model (not an ABM) to make your point. There has been much discussion in the social simulation modeling community around descriptive vs simple models. What is now a widely recognized feature of ABM is the fact that they do not need to be limited to simple rules, agents, or environments. Quite the contrary, a strong point of using these models is the capacity to generate complex systems. It was Moss and Edmonds (2005) who first popularized the idea that one not only could but should exploit the more descriptive features of ABM. These ideas reflect on the various modeling purposes (Edmonds et al. 2019) in the sense that the intensity of description may depend on the general aim of the model. In short, I believe the picture is much more nuanced than what you hint at in the paragraph mentioned at the beginning of this point.

   Edmonds B, Moss S (2005) From KISS to KIDS—an 'anti-simplistic' modelling approach. Lecture Notes in Artificial Intelligence. In: Davidson P (ed) Multi agent based simulation, vol 3415. Springer, New York, pp 130–144.

   Edmonds B, Le Page C, Bithell M, Chattoe-Brown E, Grimm V, Meyer R, Montañola-Sales C, Ormerod P, Root H, Squazzoni F (2019) Different modelling purposes J Artif Soc Soc Simul 22(3):6.

6. You do a good job in succinctly explaining the logic of a simple diffusion model. At the same time, I was wondering why you have decided to assume that one of the two individuals in the example already holds a belief. This is a slightly more complicated case than the case where the recipient does not actually have prior beliefs. This does not mean that there is going to be a necessary 'contagion' but that the likelihood for that to happen is entirely based on a probability (the probability to 'meet' this belief) without any given prior. That is more suitable with the analogy of the disease, since one does not necessarily have antibodies (i.e. the prior), and the current pandemic is a sad reminder of this. Moreover, in diffusion of innovation studies — I keep mentioning it because this has been the benchmark for diffusion research for a long time — typically there is no prior, otherwise the innovation would not be innovative. In short, I think you should at least mention that you are keeping the exemplification closer to your approach and model rather than being closer to what most equation-based models have done in the past to cover simple diffusion.

7. There are two points that came to mind as I was reading on page 6:

7.1. I follow your reasoning and it makes intuitive sense but I am not sure a model — especially after the claims you make on cognition — should be based on sole intuition. What is the theory behind the assumption that individuals would hold beliefs and be more or less prone to accept advice, recommendations, or information coming from others? This is a very important aspect that should motivate the way you model a computational simulation, especially when approaching ABM. Again, the point is not about the logic behind your modeling, but the justification of your assumptions. In my models, I use something very similar to this to justify individual dispositions towards others, and that is a version of Simon's concept of *docility* (1993). I usually frame this as a behavioral aspect of distributed cognitive processes.

Simon HA (1993) Altruism and economics. Am Econ Rev 83(2):156–161.

7.2. The other comment is more technical. At one point, you mention that you have used a way of coding strong/weak beliefs in a way that resembles a Likert scale. This is a justification that I have also used myself several times, especially at conferences to justify why certain variables were programmed the way they were. The problem I see with your way of interpreting this assertion is that you are being too literal. Psychometric scales are almost never measured on a single item, hence you never have discrete values, but a pseudo-continuous set of values for each individual that is derived from summating or averaging the score in the various items. Hence, either you are following standard measurement in cognitive psychology (using scales, not items) or you are doing something else (using discrete values). A minor point —irrelevant for the calculations — is that 7-point Likert scales are coded from '1' to '7' not from '0' to '6'.

8. As I keep reading the description of your model, I really cannot understand your claim of novelty as far as agents are kept holding heterogeneous beliefs (and dispositions) in an ABM. What you write seems standard bounded rationality assumptions for heterogeneous agents in a computational simulation model of a complex system. See the introduction of Edmonds E, Meyer R (2017). Simulating social complexity. A handbook. Springer.

9. In your formula for a binary belief update function, you assume that the positive or negative distance from one's own belief should be treated equally. However, we know that the sign of the distance is probably relevant, as one may infer from people's understanding of losses and gains in famous experiments such as those by Kahneman and Tversky (1979). You may want to argue as of why it makes sense to take the absolute value of the difference in Eq. 4.

Kahneman D, Tversky A (1979) Prospect theory: an analysis of decision under risk. Econometrica 47(2):263–292.

10. Eq. 5 is, as you explicitly state, very vague and there is a need to justify beta:

10.1. I am not sure why you have lost the 'v' in your reference belief, as if you are now treating the belief to which one is exposed as a-social or not coming from a source of any kind. Whether a belief spreads from a family member, a friend, social media, or the news, it still comes from someone. I am not sure why this turn to 'objective' belief as opposed to the one anchored to another agent — i.e. the model you have used thus far. Obviously, I would have some problems with the "objectivization" of beliefs.

10.2. I have the impression that everything that has been discussed thus far is instrumental to presenting beta and Eq. 5. If this is the case, then you could probably re-organize the

section and do it more straightforwardly, perhaps being more explicit about the quest for a beta function that captures cognition involved in misinformation spreading, to some extent.

11. From the visualization of your model, it seems that you have not created another "level" or layer (i.e. the institution) but another agent in the system. I mean, technically, this is what it is — that you have two different types of agents, one is to represent an individual (cognizer) and the other is there to represent a news outlet (an institution). Of course, you can interpret this to be two different layers but the programming does not necessarily indicate that this is the case. This means that a modeler reading your article would need a more detailed description as of why this two-agent strategy is actually identifying two layers. I do not think this is too difficult, as I have done it in some of my models—it just needs a more substantial justification.

12. As I read this section, I have noticed that you state that ABM do not often model "levels". I believe this depends on which field you are looking at. For models in economics, for example, it is customary to model a market and its agents. For models in organizational research, this is also something that happens quite often. This is related to the ability that ABM has to allow for the modeling of fast and slow timescales or, at least, this is the explanation that I think is the most valuable. You could have a look at Neumann M, Cowley SJ (2016). Modeling social agency using diachronic cognition: learning from the Mafia. In Agent-Based Simulation of Organizational Behavior (pp. 289-310). Springer, Cham.

13. The presentation of the model does not seem to follow any of the known protocols or schemes that are found in specialized journals for ABM such as, for example, the Journal of Artificial Societies and Social Simulation (JASSS). I am referring to the good custom to show, for example, a flow chart summarizing the processes, and to split the description of agents and their characteristics from the processes of the model. You may want to add a table where you define each parameter, their notation, values, experimental values, and a short description. While published articles do not include the entire ODD protocol (see Grimm et all 2020 for the latest version), it is good to include some of it.

    Grimm V, Railsback SF, Vincenot CE, Berger U, Gallagher C, DeAngelis DL, Edmonds B, Ge J, Giske J, Groeneveld J, Johnston AS (2020). The ODD protocol for describing agent-based and other simulation models: A second update to improve clarity, replication, and structural realism. Journal of Artificial Societies and Social Simulation 23(2):7.

14. One of the aspects that characterizes ABM research is that these simulations lean (more or less) heavily on stochastic components. From your presentation of the model, I am not sure I have a clear understanding of where exactly are the stochastic components of the model. Of course, a limited part of these components suggests that, perhaps, ABM may not be the preferred choice for a computational simulation.

15. The three conditions (p.9) you describe for the institutional distribution of messages are interesting and make sense from a computational/technical point of view, because you want to understand the impact of a variety of messages. At the same time, they should reflect conditions that can be observed in an actual institutional environment, unless the simulation wants to be a theoretical (in principle) case. Given the actuality of your topic, I do not think this latter is the case and so, I would ask if you could provide some example of what these conditions refer to.

16. I am not sure it is standard practice in ABM to write hypotheses (p.10) as a way to present the expectations on what to find. However, if you must, then you should at least (a) ground them in the literature and (b) explicitly formulate hypotheses. I would prefer that you are not explicit on hypotheses since, if that is the case, then formal testing needs to be performed and that would probably take the manuscript a bit far from what you intend to do.

17. When you write about “preliminary experiments” (p.10, line 341) to find the values of the threshold and the contagion probability, what is it exactly that you did? Did you perform a sensitivity analysis? And how come a constant is the outcome of such an analysis. ABM usually provide a range of values for their parameters.

18. If I understand what you write about the model and your intention is to test the effect of different graph types, I am not sure why you have decided to go with ABM as opposed to a more classic social network analysis. This point, in connection with the one on stochasticity above, needs further (better) explanations.

19. Also, the determination of the number of runs for each configuration of parameters seem very vague. You write you set on 10 without providing any detail about what procedure you followed. This is a very important aspect to understand whether results are valid.

20. Results are overall well presented and described. I have only two comments:

    20.1. The first impression is that you are trying to do too much with just one paper. There are many conditions that you want to test with this simulation and results are rich. However, too much information is probably as dangerous as its paucity. Maybe there is a way to limit the amount of graphs and comments and this could be done with a more structured systematic approach to the findings. You can start by presenting a table where computational experiments are classified and indicate the main finding for each one of them. Some results are similar and you could skip them, unless significant differences appear in the configuration tested. You may want to merge the presentation of results and their analysis, where some of these summary tables already appear. Given these tables, I am not sure whether the many graphs provide additional information.

    20.2. The second comment is about the uncharacteristic form that most of your graphs show in relation to the type of simulation modeling you have conducted (ABM). This points at the minimal stochasticity that seems to be embedded in your model. I may be too much leaning towards models with a large stochastic component, but linear results such as the ones that most of your results show require an explanation or, maybe, just a more straightforward presentation of the ABM.

21. I think I can stop here with my comments and come back to the final part of the paper once you have addressed the above.
