# Supplementary material for: Cognitive cascades: How to model (and potentially counter) the spread of fake news
Source: PLoS One. 2022 Jan 7;17(1):e0261811. doi: 10.1371/journal.pone.0261811 (PMC8740964; doi:10.1371/journal.pone.0261811)
Supplement: Attachment — Submitted filename: Review of PONE-D-21-06938.R1.pdf [file pone.0261811.s032.pdf]

Review of PONE-D-21-06938.R1: Cognitive cascades: How to model (and potentially counter) the spread of fake news

Thank you very much for your hard work on the manuscript, I think the revision has improved it significantly from the previous version. I have enjoyed reading this version as well as the previous one and I believe it makes an important contribution to the literature. As an agent-based modeler, I am still slightly uncomfortable with some of your choices concerning modeling and their reporting on the paper. However, most of my concerns have been addressed and I am more comfortable with this version of the paper.

There are a few open points that you may want to address before submitting the paper in its final version. I have reported them below — some refer to previous comments that require additional attention while others are new. I assess these comments as requiring minor efforts on your part.

31. **Former point 3**. I think your revised introduction clarifies the scope and objectives of the paper well — much better than before. One point that I think still requires your attention is the definition of a cognitive 'cascade'. In fact, I cannot find a place where you define what you mean with this. You simply write that cognitive cascade models are "those that adopt a cascading network-based diffusion model from social network theory" (p.2, lines 26-27). Fine, but what is the cascade in a cascading network-based model? It seems you define cascading models through cascading networks. The result is a very unclear definition to those who do not know the meaning of a cascade.

32. **Former point 5**. When you refer to ABM in the social sciences you still refer to Schelling's cellular automata model [48]. My comment was probably unclear on this point, since I was also trying to make another point (which you addressed). Apologies for repeating but [48] is not an ABM. You can cite any ABM published in JASSS to make your point or refer to the classic books by Gilbert, Edmonds, Troitzsch, etc. Here are some of them:

    Edmonds E, Meyer R (2017). *Simulating social complexity*. A handbook. Springer.

    Gilbert, N. (2019). *Agent-based models* (Vol. 153). Sage Publications.

    Gilbert, N., & Troitzsch, K. (2005). *Simulation for the social scientist*. McGraw-Hill Education (UK).

33. **Former point 7.2**. I have mentioned the use of Likert scale before and the fact that I find this approach very much in line with how modelers reason about numerical representations of beliefs. I do not quite understand your reply though. To my claim that you have been too literal and used a discrete value approach rather than mimicking the use of a Likert scale you have replied by adding a sensitivity analysis of the values of parameter *b*. I understand you performed those checks as reply to a point from another reviewer but that does not address my point. Please do let me know if I am missing something here. Do not get me wrong, I do think that your approach is fine; it is just that your claim is not fully consistent with the use of a Likert scale in applied psychology.

34. **Former point 9**. I could not find your response in the text of the article. I think it is a convincing one, please go ahead and find room for it.

35. **Former point 14**. I appreciate your reply and it may be a good idea to specify these points somewhere in the paper.

36. **Former point 16**. I respect your choice of leaving hypotheses but then I would expect them being accepted or rejected following some more formal testing procedure. Another way to do this would be to call them differently (e.g., assumptions, propositions) so that a reader does not expect formal testing. Now, the test you have used is correlation (I could see Chi-square tests from some of your tables as well). You may want to use that to test your hypotheses although it is a test of association, not one that would help you understanding whether the spread (or lack thereof) was determined to some extent by the conditions of the simulation. Maybe a test where you can actually show dependence between conditions and output would probably make your point stronger.

37. **Former point 19**. I have read your supplementary materials files and I am afraid I was not able to find a justification for the 10 repetitions. In one of these files you state “We varied *p*, the contagion probability, from 0.05 to 0.95 in increments of 0.05, and took averages over 10 simulation runs”. This means that 10 was a given. “Why 10” and not 9 or 13, 57 or 9762 was my question.

38. **Former point 20.1**. I still think that you can find a better — more succinct way — of presenting your results. There are currently 14 figures in the paper and these are probably too many. Unless you convincingly argue that they all have something unique to tell about your results, I would consider moving some to the supplementary materials. Another way to deal with this is that of presenting results together with their analysis. You do not need to describe and then analyze, you can describe as you analyze results. I appreciate you do the analysis a bit more precisely, but some graphs can be made to compare results already and differences could be shown as a comment to those graphs.

39. A minor point: In the introduction you write about social network theory and cognition theory. I believe you want to refer to social network research (or analysis) and cognitive science. In particular, the latter (i.e. “cognition theory”) does not exist although there are many cognition theories that belong to the different domains of cognitive science. I suggest you make the reference to theories in the plural or change them accordingly.

40. If you are mainly referring to the status of misinformation in the US, you should explicitly state it at the beginning of the article, when you bring in the first example. If you do not do that, the problem is that you assume readers implicitly think of the context as the US when, in reality, an article can be read from anywhere in the world. Maybe using the US as a reference has its advantages in the sense that misinformation seems to have reached particularly concerning levels there as compared to other countries. [I am using vaccination rates as a proxy and numbers are alarming if one compares the US with, say, Spain or the European Union as a whole.] So, please, try to be less US-centric and inform readers that you are taking this perspective.

41. I appreciate the new focus on cognitive cascade models although you keep swinging between contagion and cascade (pp.5f). It may be a good idea to clarify the connection between the two and, at the same time, try and be more consistent in the use of terminology throughout the paper.

42. Continuing from the point above, at page 6 you give the impression that the cascade is an event (“during a cascade”, line 196). This reminds me of the fact that you still have not defined what a cascade is. Something close to a definition comes much later (p.8).
