# Supplementary material for: Cognitive cascades: How to model (and potentially counter) the spread of fake news
Source: PLoS One. 2022 Jan 7;17(1):e0261811. doi: 10.1371/journal.pone.0261811 (PMC8740964; doi:10.1371/journal.pone.0261811)
Supplement: Attachment — Submitted filename: reviewers-response.pdf [file pone.0261811.s033.pdf]

(Our responses in blue)

We would like to thank all reviewers for their comments and suggestions, which we believe again improved our revised draft of the paper. Specific responses to individual questions from reviewers can be found below.

Reviewer 1: N/A

Reviewer 2: Thank you for responding so thoroughly to my previous comments. The only area of outstanding concern for me is in the sensitivity test to the number of levels in your model. In my previous review, I asked that you check that the conclusions of your model are not sensitive to the arbitrary number of levels you chose. In theory, if the results are rigorous they should hold equally well despite the number of levels. Thank you for conducting this sensitivity test and reporting the results.

I am worried however that your tests did not find that your conclusions were truly robust to the number of levels in the model. In your response and supplement you state this, but in the paper itself you merely say that you ran a sensitivity test. The main body of the paper makes no mention of the fact that the results seem to be strongly influenced by the discretization assumption. In fact, the sentence "We additionally ran in-silico experiments with lower and higher resolutions..." seems to imply that you didn't see any difference by going to more continuous levels of belief, especially as you had previously said "bu can be a continuous variable with the interval from strong disbelief to strong belief, or it can take on discrete values". At the very minimum, you have a disconnect between the scope condition you are claiming (continuous levels belief) and the domain over which your model predicts the outcome you claim (discrete belief levels). To be completely honest with your readers, I think more of this discussion of the reliance on discrete belief levels belongs in your main text. Don't let anyone suspect you're hiding anything.

Thank you for this point, we agree. We have added language to clarify that while we can model things that approach continuity, that for some of our experiments, the dynamics became different when the number of different “belief levels” became large enough (while still showing there is nothing magic about 7 and that slightly fewer or more but still discrete belief states gave similar network dynamics.) We suspect this is a limit of the way we set initial model parameters during experiments, more than something intrinsically different about true continuous belief states, but we now make it very clear that there’s a difference in the main paper.

What your discretization assumption seems to be doing is acting as a coarse proxy for similarity between neighbors, so that you don't have to justify a rule for who individuals pay attention to that works in the continuous domain. As your results are dependent on this, you need to be more explicit

about it. Otherwise, you can update your model to allow for similarity given a continuous measure of belief, and see if your results still hold. This would be a more robust result, and easier for you to justify if it doesn't add too much modeling complexity.

Completely agree – see above.

Reviewer 3:

Thank you very much for your hard work on the manuscript, I think the revision has improved it significantly from the previous version. I have enjoyed reading this version as well as the previous one and I believe it makes an important contribution to the literature. As an agent-based modeler, I am still slightly uncomfortable with some of your choices concerning modeling and their reporting on the paper. However, most of my concerns have been addressed and I am more comfortable with this version of the paper. There are a few open points that you may want to address before submitting the paper in its final version. I have reported them below —some refer to previous comments that require additional attention while others are new. I assess these comments as requiring minor efforts on your part.

31.Former point 3. I think your revised introduction clarifies the scope and objectives of the paper well —much better than before. One point that I think still requires your attention is the definition of a cognitive 'cascade'. In fact, I cannot find a place where you define what you mean with this. You simply write that cognitive cascade models are "those that adopt a cascading network-based diffusion model from social network theory" (p.2, lines 26-27). Fine, but what is the cascade in a cascading network-based model? It seems you define cascading models through cascading networks. The result is a very unclear definition to those who do not know the meaning of a cascade.

We now define "cascades" explicitly in the introduction, and contrast it to how we intend to use the word "contagion."

32.Former point 5. When you refer to ABM in the social sciences you still refer to Schelling's cellular automata model [48]. My comment was probably unclear on this point, since I was also trying to make another point (which you addressed). Apologies for repeating but [48] is not an ABM. You can cite any ABM published in JASSS to make your point or refer to the classic books by Gilbert, Edmonds, Troitzsch, etc. Here are some of them:

- Edmonds E, Meyer R (2017). Simulating social complexity. A handbook. Springer.
- Gilbert, N. (2019).Agent-based models(Vol. 153). Sage Publications.
- Gilbert, N., & Troitzsch, K. (2005).Simulation for the social scientist. McGraw-Hill Education (UK).

We have now changed out the Schelling 1971 reference to the two references you provided from Gilbert.

33.Former point 7.2. I have mentioned the use of Likert scale before and the fact that I find this approach very much in line with how modelers reason about numerical representations of beliefs. I do not quite understand your reply though. To my claim that you have been too literal and used a discrete value approach rather than mimicking the use of a Likert scale you have replied by adding a sensitivity analysis of the values of parameter b. I understand you performed those checks as reply to a point from another reviewer but that does not address my point. Please do let me know if I am missing something here. Do not get me wrong, I do think that your approach is fine; it is just that your claim is not fully consistent with the use of a Likert scale in applied psychology.

By showing that the model is somewhat robust to whether a 7- or 5- or 9-point scale is used (where we indeed used 7 as inspired by Likert scales), we hoped that this would dissuade any interpretation of our choice of 7 points as trying to exactly emulate Likert scales. Hopefully we have removed any indication that we are exactly using the Likert scale the way it is used in applied psychology.

34.Former point 9. I could not find your response in the text of the article. I think it is a convincing one, please go ahead and find room for it.

We wrote language after Eq. 4 to capture what we wrote in the previous comments.

35.Former point 14. I appreciate your reply and it may be a good idea to specify these points somewhere in the paper

Thank you. We captured this now at the end of the "Experimental Design" subsection.

36. Former point 16. I respect your choice of leaving hypotheses but then I would expect them being accepted or rejected following some more formal testing procedure. Another way to do this would be to call them differently (e.g., assumptions, propositions) so that a reader does not expect formal testing. Now, the test you have used is correlation (I could see Chi-square tests from some of your tables as well). You may want to use that to test your hypotheses although it is a test of association, not one that would help you understanding whether the spread (or lack thereof) was determined to some extent by the conditions of the simulation. Maybe a test where you can actually show dependence between conditions and output would probably make your point stronger.

In order to be less confusing, we have changed the word "hypothesis" or "hypothesize" to "we expect" or "we anticipate" that do not imply that we will conduct statistical tests to test those hypotheses.

37.Former point 19. I have read your supplementary materials files and I am afraid I was not able to find a justification for the 10 repetitions. In one of these files you state “We varied p, the contagion probability, from 0.05 to 0.95 in increments of 0.05, and took averages over 10 simulation runs”. This means that 10 was a given. “Why 10” and not 9 or 13, 57 or 9762 was my question.

Thank you for catching this. We agree that our previous answer did not address that aspect. We have run additional correlation tests and included a write-up of results in supplementary material S1 Text.

38.Former point 20.1. I still think that you can find a better —more succinct way —of presenting your results. There are currently 14 figures in the paper and these are probably too many. Unless you convincingly argue that they all have something unique to tell about your results, I would consider moving some to the supplementary materials. Another way to deal with this is that of presenting results together with their analysis. You do not need to describe and then analyze, you can describe as you analyze results. I appreciate you do the analysis a bit more precisely, but some graphs can be made to compare results already and differences could be shown as a comment to those graphs.

Is there something we can move to the supplement? Doesn’t hurt.

We agree that this was a verbose way to present our results. During our presentation of results under the section “Comparing Contagion Methods,” we have removed some of the superfluous description of results, as well as moved the simple and proportional threshold cascade results to the supplements.

39.A minor point: In the introduction you write about social network theory and cognition theory. I believe you want to refer to social network research (or analysis) and cognitive science. In particular, the latter (i.e. “cognition theory”) does not exist although there are many cognition theories that belong to the different domains of cognitive science. I suggest you make the reference to theories in the plural or change them accordingly.

We have changed “cognitive theory” to simply say “cognitive science” instead to avoid confusion.

40.If you are mainly referring to the status of misinformation in the US, you should explicitly state it at the beginning of the article, when you bring in the first example. If you do not do that, the problem is that you assume readers implicitly think of the context as the US when, in reality, an article can be read from anywhere in the world. Maybe using the US as a reference has its advantages in the sense that misinformation seems to have reached particularly concerning levels there as compared to other countries. [I am using vaccination rates as a proxy and numbers are alarming if one compares the US with, say, Spain or the European Union as a whole.] So, please, try to be less US-centric and inform readers that you are taking this perspective.

This is a welcome point, thank you for bringing it to our attention. We have added explanations that we are primarily focusing on evidence from the U.S., even though COVID misinformation appears in many countries. We now make this more clear in the introduction.

41.I appreciate the new focus on cognitive cascade models although you keep swinging between contagion and cascade (pp.5f). It may be a good idea to clarify the connection between the two and, at the same time, try and be more consistent in the use of terminology throughout the paper.

Thank you for catching the inconsistencies. We now consistently use the language of “cascades” where applicable, and when mentioning “contagion,” make clear the difference, as we define it, between individual-level contagion and macro-level cascades.

42.Continuing from the point above, at page 6 you give the impression that the cascade is an event (“during a cascade”, line 196). This reminds me of the fact that you still have not defined what a cascade is. Something close to a definition comes much later (p.8).

As with the above point 31, we now define cascades explicitly in the introduction and differentiate it from contagion.
